# Supplementary material for: Site-specific acetylation of polynucleotide kinase 3′-phosphatase regulates its distinct role in DNA repair pathways
Source: Nucleic Acids Res. 2024 Jan 15;52(5):2416–33. doi: 10.1093/nar/gkae002 (PMC10954452; doi:10.1093/nar/gkae002)
Supplement: gkae002_Supplemental_File [file gkae002_supplemental_file.pdf]

Supplementary Figure 1

(A)

PPM	XCorr	ΔCorr	Peptide	Target amino acid
0.79	3.422	0.555	R.**K#**SNPGWENLEK.L	K142
0.92	2.091	0.694	R.G**K#**LPAEEFK.A	K226

(B)

Predicted Fragmentation Pattern

Seq	#	b: Δ Error	b	y	y: Δ Error	+1
K#	1	---	171.1	---	---	11
S	2	-0.1	258.1	1173.6	0.1	10
N	3	0.0	372.2	1086.5	0.0	9
P	4	0.2	469.2	972.5	0.0	8
G	5	0.0	526.3	875.4	0.0	7
W	6	0.0	712.3	818.4	0.1	6
E	7	0.0	841.4	632.3	0.0	5
N	8	0.3	955.4	503.3	0.0	4
L	9	-0.1	1068.5	389.2	0.1	3
E	10	0.0	1197.6	276.2	0.2	2
K	11	---	---	147.1	---	1

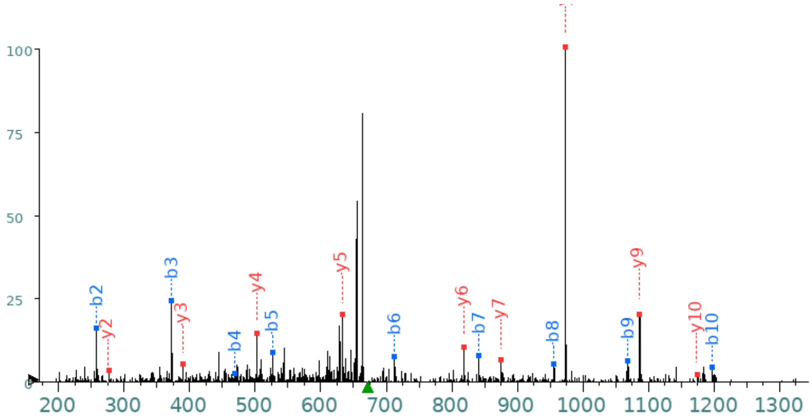

(C)

Predicted Fragmentation Pattern

Seq	#	b: Δ Error	b	y	y: Δ Error	+1
G	1	---	58.0	---	---	9
K#	2	0.1	228.1	1003.5	---	8
L	3	0.0	341.2	833.4	0.2	7
P	4	0.0	438.3	720.4	0.0	6
A	5	0.0	509.3	623.3	0.1	5
E	6	0.2	638.4	552.3	0.3	4
E	7	0.0	767.4	423.2	0.1	3
F	8	0.1	914.5	294.2	0.1	2
K	9	---	---	147.1	---	1

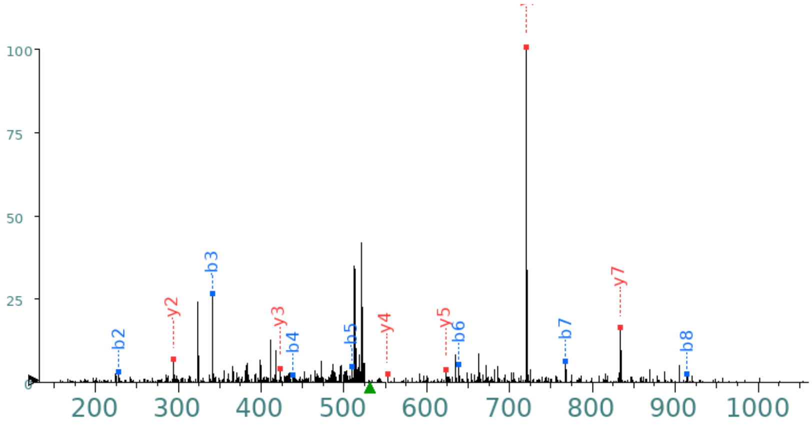

Supplementary Figure 1 (contd)

(D)

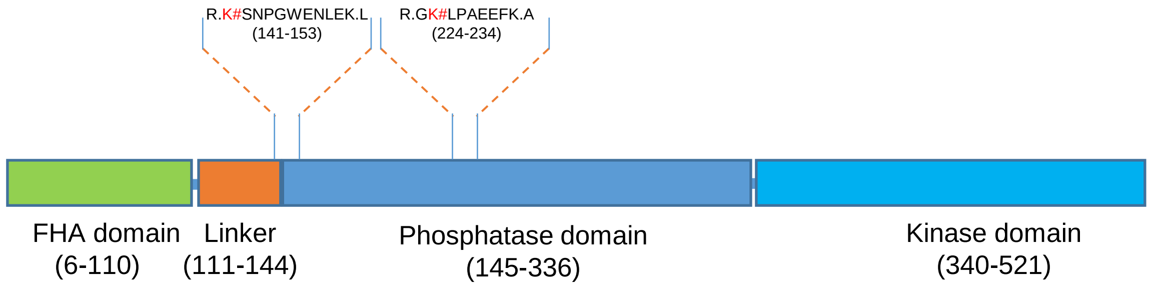

(E)

PNKP	Treatment	Acetylated amino acid #
WT-FLAG	Mock	K142
WT-FLAG	(+) Bleo	K142/K226
K226R-FLAG	Mock	K142
K226R-FLAG	(+) Bleo	K142

Supplementary Figure 1. Identification of two novel acetyl lysine sites in PNKP by LC-MS/MS. (A) Acetylation sites are marked in red within the peptide sequence and the amino acid residues are listed in a subtitle as well. The table shows the Sequest algorithm scores (Xcorr and ΔCorr) along with the mass accuracy measurement of the acetylated peptides. (B and C) The figures show the fragmentation pattern of two acetylated peptides matched to PNKP. Representative figures of acetyl-lysine spectrums of PNKP are shown: (B) the spectrum of peptide sequence for K142 acetylation and (C) the spectrum of peptide sequence for K226 acetylation**.** The acetylated lysines are shown as K#. The # symbol represents a mass addition of 42.0106 Da to lysine. (D) Schematic representation of PNKP domains (FHA, Linker, phosphatase and kinase), indicating the K142 acetylation site in the linker region and the K226 acetylation site in the phosphatase domain. (E) Results of a similar Mass Spectrometric analysis are represented in the table showing the acetylated residues in WT vs. K226R PNKP stable cell lines ±Bleo.

Supplementary Figure 2

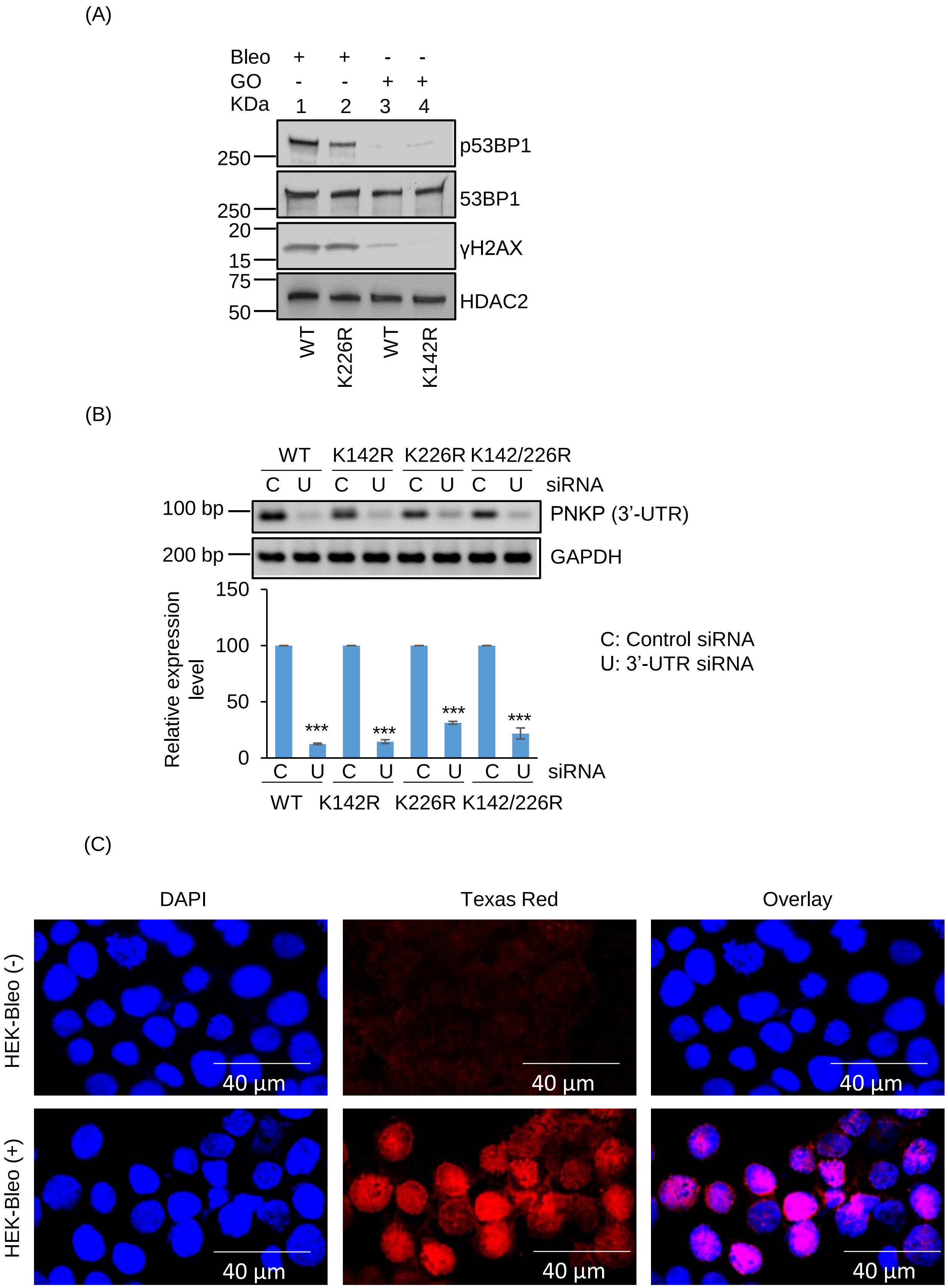

Supplementary Figure 2. (A) Western blot shows the expression level of phosphorylated 53BP1, total 53BP1 and γH2AX in the chromatin fraction of Bleo-treated WT (lane 1) and K226R (lane 2) and GO-treated WT (lane 3) and K142R (lane 4) cells. HDAC2: used as a loading control. (B) Depletion of endogenous PNKP by 3'-UTR specific siRNA. The representative agarose gel shows the extent of depletion of endogenous PNKP in WT, K142R, K226R and K142R/226R PNKP expressing stable cell lines by 3'UTR specific siRNA. GAPDH was used as housekeeping control (upper panels). The bar diagram represents the relative expression level of endogenous PNKP normalized with the expression of control GAPDH with the control siRNA transfected samples considered as 100 (Error bars represent ±SD of the mean, n=3, ***P<0.005) (lower panel). (C) Mock- or Bleo-treated WT HEK293 were stained with an anti-AcK226 Ab. Nuclei were counterstained with DAPI.

Supplementary Figure 3

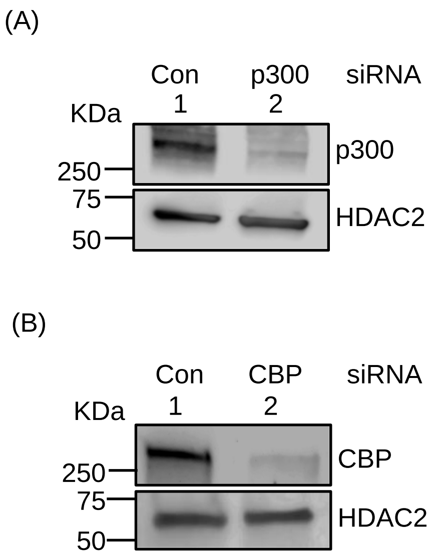

Supplementary Figure 3. Depletion of endogenous p300 and CBP by siRNA in HEK293 cells. Western blots show the relative expression level of (A) p300 and (B) CBP in the chromatin fraction following control (Con; lane 1) and specific siRNA (lane 2) transfection. HDAC2 is used as a loading control.

Supplementary Figure 4

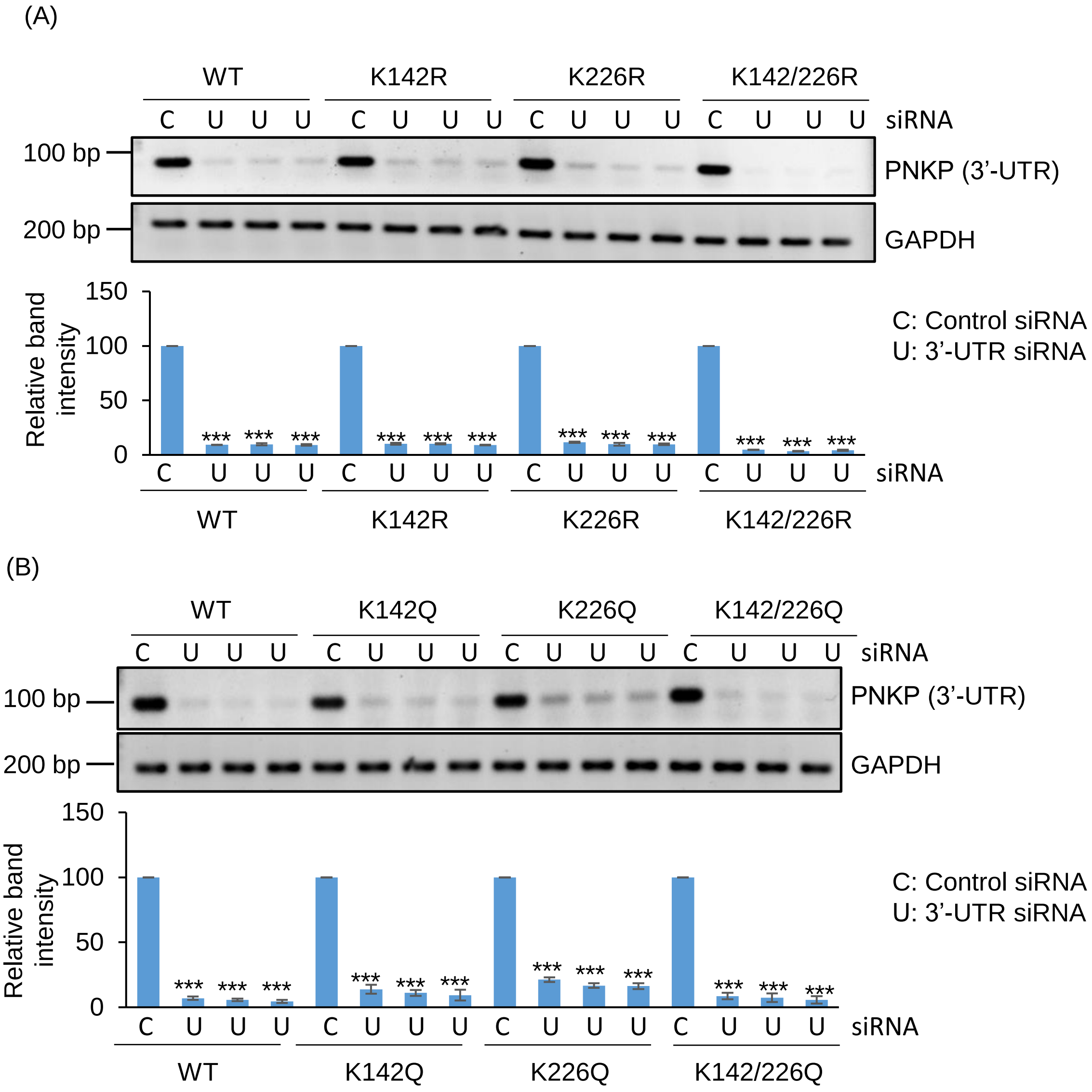

Supplementary Figure 4. Depletion of endogenous PNKP by 3'-UTR specific siRNA. The representative agarose gels (upper panels) show the extent of depletion of endogenous PNKP in WT, K142R, K226R and K142R/226R PNKP expressing stable cell lines (A) and WT, K142Q, K226Q and K142Q/226Q PNKP expressing stable cell lines (B) by 3'UTR specific siRNA. The bar diagrams represent the relative expression level of endogenous PNKP normalized with the expression of control GAPDH with the control siRNA transfected samples considered as 100 (Error bars represent ±SD of the mean, n=3, ***P<0.005) (lower panels).

Supplementary Figure 5

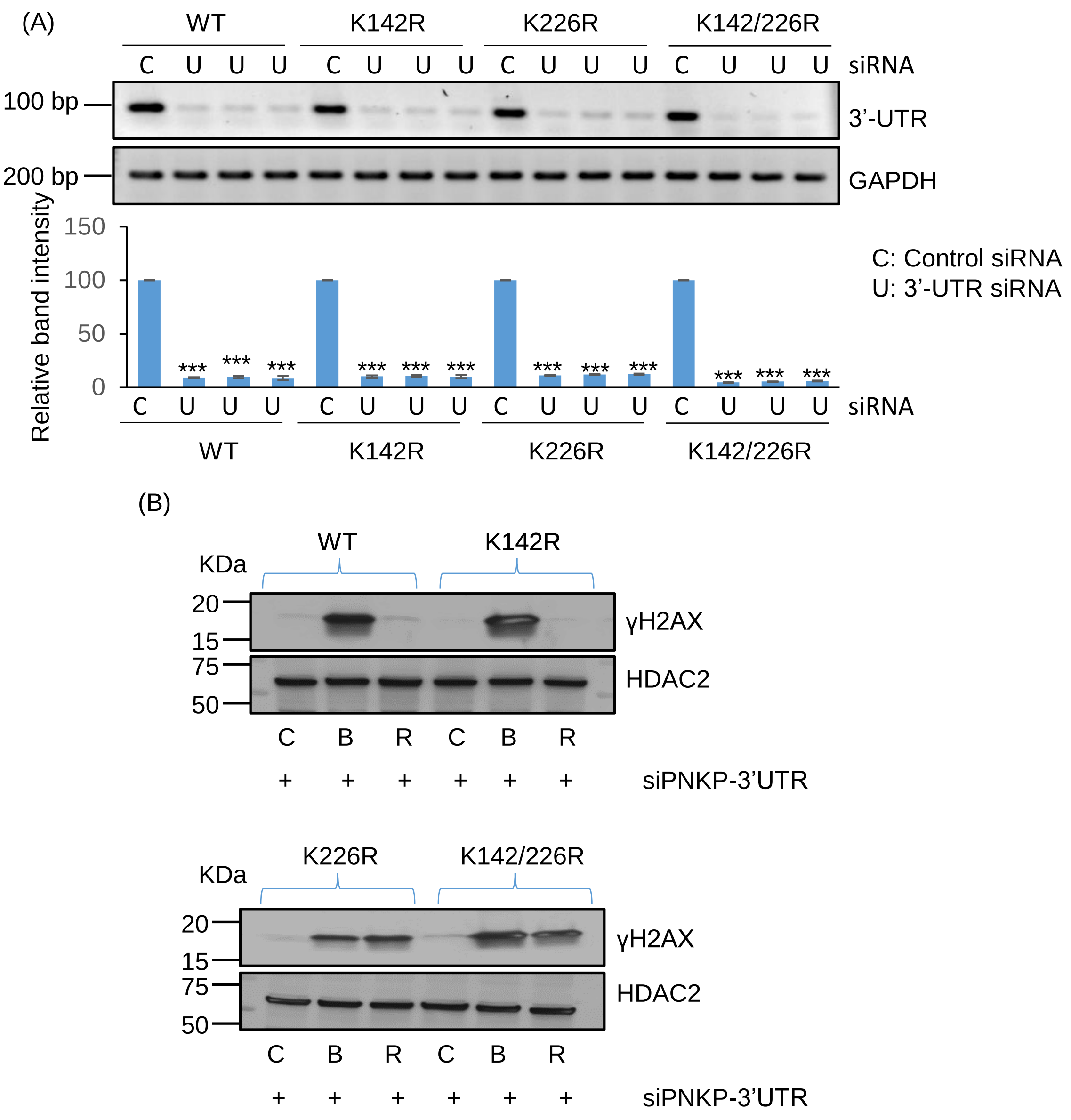

Supplementary Figure 5 (contd)

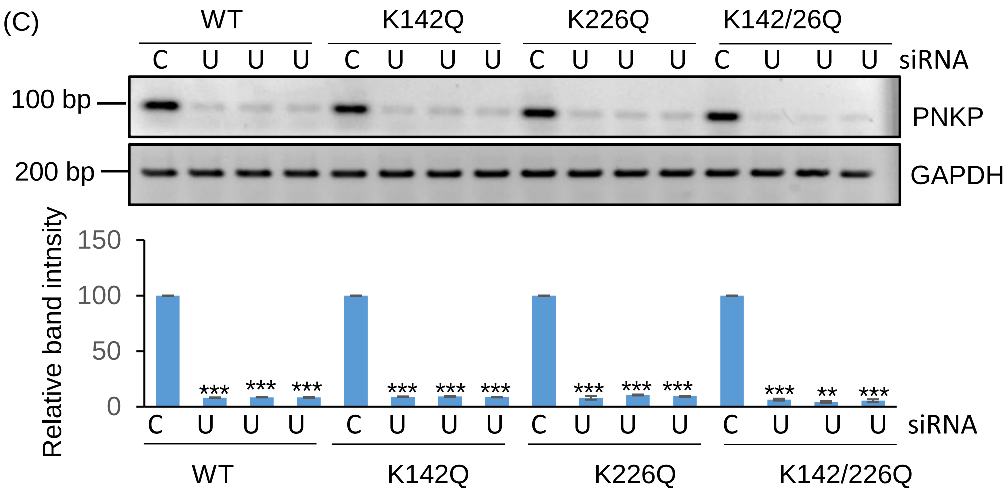

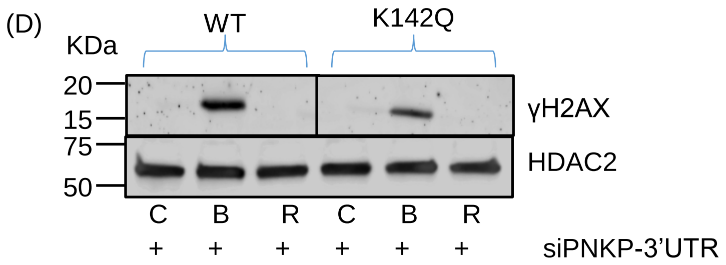

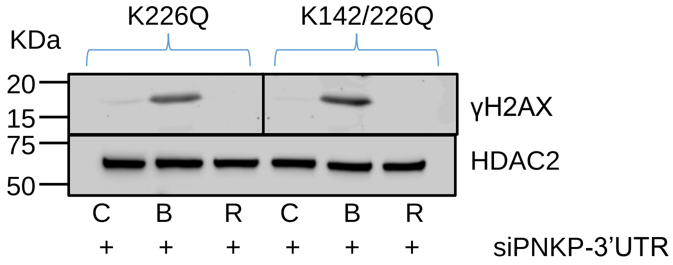

Supplementary Figure 5. (A and C) (Upper panels) The representative agarose gels show the extent of depletion of endogenous PNKP in (A) WT, K142R, K226R and K142R/226R PNKP expressing stable cell lines and in (C) WT, K142Q, K226Q and K142Q/226Q PNKP expressing stable cell lines under the same experimental conditions. (Lower panels) The bar diagrams represent the relative expression level of endogenous PNKP normalized with the expression of control GAPDH with the control siRNA transfected samples considered as 100 (Error bars represent ±SD of the mean, n=3, ***P<0.005). (B and D) Western blots show the expression of γH2AX (upper panels) in the chromatin fraction of (B) WT, K142R, K226R and K142R/226R PNKP expressing cells and (D) WT, K142Q, K226Q and K142Q/226Q expressing cells mock (C), Bleo-treated (B) or 16 h post Bleo-treatment (R). HDAC2 is used as the loading control (lower panels).
